# Host-microbiota interaction-mediated resistance to inflammatory bowel disease in pigs

**DOI:** 10.1186/s40168-022-01303-1

**Published:** 2022-07-30

**Authors:** Xuan Zhao, Lin Jiang, Xiuyu Fang, Zhiqiang Guo, Xiaoxu Wang, Baoming Shi, Qingwei Meng

**Affiliations:** Institute of Animal Nutrition, Northeast Agricultural University, Harbin, 150030 People’s Republic of China

**Keywords:** Disease resistance, Microbiota, Metabolome, Immunity response, Intestinal barrier function

## Abstract

**Background:**

Disease resistance phenotypes are associated with immune regulatory functions and immune tolerance and have implications for both the livestock industry and human health. Microbiota plays an essential role in regulating immunity and autoimmunity in the host organism, but the influence of host-microbiota interactions on disease resistance phenotypes remains unclear. Here, multiomics analysis was performed to identify potential regulatory mechanisms of disease resistance at both the microbiome and host levels in two pig breeds.

**Results:**

Acute colitis models were established in Min pigs and Yorkshire pigs, and control and diseased individuals were compared. Compared with Yorkshire pigs under the same nutritional and management conditions, Min pigs exhibited strong disease resistance, as indicated by a low disease activity index (DAI) and a low histological activity index (HAI). Microbiota sequencing analysis showed that potentially harmful microbes *Desulfovibrio*, *Bacteroides* and *Streptococcus* were enriched in diseased individuals of the two breeds. Notably, potentially beneficial microbes, such as *Lactobacillus*, *Clostridia* and *Eubacterium*, and several genera belonging to *Ruminococcaceae* and *Christensenellaceae* were enriched in diseased Min pigs and were found to be positively associated with the microbial metabolites related to intestinal barrier function. Specifically, the concentrations of indole derivatives and short-chain fatty acids were increased in diseased Min pigs, suggesting beneficial action in protecting intestinal barrier. In addition, lower concentrations of bile acid metabolites and short-chain fatty acids were observed in diseased Yorkshire pigs, which were associated with increased potentially harmful microbes, such as *Bilophila* and *Alistipes*. Concerning enrichment of the immune response, the increase in CD4^+^ T cells in the lamina propria improved supervision of the host immunity response in diseased Min pigs, contributing to the maintenance of Th2-type immune superiority and immune tolerance patterns and control of excessive inflammation with the help of potentially beneficial microbes. In diseased Yorkshire pigs, more terms belonging to biological processes of immunity were enriched, including Toll-like receptors signalling, NF-κB signalling and Th1 and Th17-type immune responses, along with the increases of potentially harmful microbes and damaged intestinal barrier.

**Conclusions:**

Cumulatively, the results for the two pig breeds highlight that host-microbiota crosstalk promotes a disease resistance phenotype in three ways: by maintaining partial PRR nonactivation, maintaining Th2-type immune superiority and immunological tolerance patterns and recovering gut barrier function to protect against colonic diseases.

Video abstract.

**Supplementary Information:**

The online version contains supplementary material available at 10.1186/s40168-022-01303-1.

## Background

The gut harbours a diverse community of microbiota that exhibit large between-individual variations [1], which next-generation sequencing has linked to host phenotypes [2]. Millions of years of coevolution between host and microbiota have led to a mutualistic relationship in which microbiota likely mediate host physiological suitability [3]. Specifically, host physiology can be altered by microbiota-induced cell signalling and proliferation, leading to changes in the mucosa, barrier function and immune response and thereby affecting phenotype [4–6]. Given the broad range of effects of microbiota on host physiology and the induction and function of the immune system, it is unsurprising that the combination of host and microbiota largely drives the development of disease resistance phenotypes. The immune response to disease is an essential indicator of the host’s disease resistance phenotype, which differs depending on microbiota composition. Host-microbiota interactions have been implicated in various gut-related diseases, including diarrhoea, enterotoxaemia and inflammatory bowel disease (IBD) [7–10]. These diseases are characterised by microbial dysbiosis and perturbations of host homeostasis, but the roles of changes in the microbiota in different disease resistance phenotypes have not been entirely determined. Moreover, whether and to what extent the gut microbiota drives diseases or is altered in response to disease remains unclear. Intervention studies targeting different disease resistance phenotypes are required to address this gap. Given the importance and complexity of microbiota across different pig breeds, there is increasing interest in identifying the composition of the microbiota and their functional contribution to achieve a better understanding of the mechanism of disease resistance phenotype formation.

The Yorkshire pig is a common commercial pig breed worldwide and is found in most countries with pig breeding industries. Pedigreed pigs of this breed are adaptable to various environments and display a certain resistance to diseases [11], and the difference in the disease resistance phenotype compared with native breeds may not be small [12]. Min pigs are a native Chinese breed with stronger disease resistance than Yorkshire pigs. Previous studies have demonstrated that compared with other breeds, Min pigs reach the peak of diarrhoea later in the weaning period and have a lower diarrhoeal burden [13]. The more excellent disease resistance of Min pigs compared with other breeds, such as Yorkshire pigs, might offer a route to characterise efficient microbiomes. Recently, we found that Min pig faecal microbiota transfer could modulate the intestinal microbiota and increase immunoglobulins and antimicrobial peptides in Yorkshire piglets [14], suggesting that the microbiota of Min pigs can enhanced the health of Yorkshire pigs by affecting host physiology. This may also partially explain why the disease resistance of Min pigs is stronger than that of Yorkshire pigs. While we have identified an association of the microbiota with diseases, how certain core bacteria in Min pigs contribute to shaping the disease resistance phenotype compared with Yorkshire pigs under the same nutrition and management remains unclear. We hypothesised that Yorkshire pigs might lack important host-microbiota interactions, resulting in weak host adaptability under pressure from diseases and inflammatory immune stimuli. DSS-induced colitis is a well-established model of acute colitis with ulceration resembling ulcerative colitis in humans [15, 16]. Ulcerative colitis, a type of IBD, can be viewed as an autoimmune disease strongly influenced by disruptions in host-microbiota homoeostasis [17]. Therefore, in this study, we employed the DSS-induced colitis model to identify certain commensal bacteria and their metabolites in Min pigs and Yorkshire pigs that may affect host physiological suitability and subsequently affect IBD susceptibility. The current study provides fundamental information about the potential links between gut microbiota and host phenotypes and may contribute to screening for core intestinal microbial candidates associated with host health.

## Methods

### Animals and experimental design

All animal procedures were performed in compliance with the approval of the Institutional Animal Care and Use Committee of Northeast Agricultural University (certification number NEAU-[2011]-9). Sixteen Min pigs (50 days old) and sixteen Yorkshire pigs (50 days old) in the same physiological state were selected from a commercial farm and individually housed in stainless steel metabolism crates with automatic troughs and drinking water nipples for free access to water. Animals received the same diet. After 10 days of acclimation, the pigs of each breed were randomised into 2 treatments administered via gavage for 5 days: CON (control, sterile saline) and DSS (4% DSS in 100 mL of water). The first dose of DSS water had a volume of 200 mL and was preceded by a 12-h fasting period. The dose of DSS was chosen by referring to Bassaganya et al. [18]. All pigs were slaughtered at 66 days of age. Samples were collected from each pig immediately after slaughter. Colon tissue was rinsed and stored immediately at −80 °C until further analysis. Colon contents were collected and stored at −80 °C until microbiota and metabolite analyses.

### Phenotypic clinical evaluation

#### Disease Activity Index (DAI)

Following DSS treatment, the disease activity index (DAI) was monitored on a daily basis. The DAI score was calculated as (body weight loss score + stool consistency score + haematochezia score)/3. The body weight loss score ranged from 0 to 3: 0 = stable; 1 = mild loss (lost > 1% to < 5%); 2 = moderate loss (lost > 5% to < 10%); and 3 = severe loss (lost > 10%). The stool consistency score ranged from 0 to 3: 0 = normal, 1 = soft, 2 = fluid and 3 = completely liquid. The haematochezia score was determined by the colloidal gold method.

#### H&E staining

The severity of colonic lesions was scored macroscopically and histologically. Colon samples were fixed in 4% paraformaldehyde, embedded in paraffin and sectioned and stained with haematoxylin and eosin (H&E) for histological examination to determine the severity of inflammation and the extent of mucosal and crypt damage. Images were captured at 100× magnification using a US Moticam 3000 photomicrography imaging system.

#### Organ index

The spleen and colon were collected and evaluated by the organ index formula: organ index (%) = (organ weight/body weight) × 100%.

### Sequencing of microbiota from colonic digesta samples and data analysis

#### DNA extraction

According to the manufacturer’s instructions, total genomic DNA was extracted from colonic digesta using the E.Z.N.A. ®Stool DNA Kit (D4015, Omega, Inc., USA).

#### PCR amplification and microbiota sequencing

The V3-V4 region of the bacterial 16S rDNA gene was amplified using the primers 341F (5′-CCTACGGGNGGCWGCAG-3′) and 805R (5′-GACTACHVGGGTATCTAATCC-3′) combined with barcode sequences. Then, the amplicon pools were prepared for sequencing, and the size and quantity of the amplicon library were assessed on an Agilent 2100 Bioanalyzer (Agilent, USA) and with the Library Quantification Kit for Illumina (Kapa Biosciences, Woburn, MA, USA), respectively. The libraries were sequenced on the NovaSeq PE250 platform.

#### Bioinformatics analysis

The sequences obtained by sequencing were subjected to quality filtering and modification. Alpha diversity and beta diversity were calculated by random normalisation to the same sequences. Then, according to the SILVA (release 132) classifier, feature abundance was normalised using the relative abundance of each sample. LEfSe was performed to assess differences in abundance. Phenotypes of bacterial communities were predicted using BugBase. The microbiome functions were determined by the KEGG profiles by combining the resulting metagenomic data with biodiversity data.

### Colonic metabolite measurements and bioinformatics analysis

The collected samples were thawed on ice, and metabolites were extracted with 50% methanol buffer. Then, all samples were analysed by LC-MS according to the instructions for the system, and a TripleTOF 5600 Plus high-resolution tandem mass spectrometer (SCIEX, UK) was used to detect metabolites eluted from the column. The online KEGG database was used to annotate the metabolites by matching the exact molecular mass data (m/z) of samples with those from a database. We used an in-house fragment spectrum library of metabolites to validate the metabolite identification. Student *t*-tests were conducted to detect differences in metabolite concentrations between the two phenotypes. The *P*-value was adjusted for multiple tests using an FDR (false discovery rate). Supervised PLS-DA was conducted to discriminate the different variables between groups. The VIP value was calculated. A VIP cut-off value of 1.0 was used to select important features. For SCFA analysis, phosphoric acid (0.5% v/v) solution was added to the colonic digesta samples, then vortexed for 10 min, ultrasonicated for 5 min and centrifuged at 12,000 r/min and 4 °C for 10 min. Then, the supernatant was collected, and MTBE (containing internal standard) solution was added. After centrifugation at 12,000 r/min for 10 min at 4 °C, the supernatant was aspirated into a sampling bottle for GC***–***MS/MS analysis.

### RNA-seq analysis from colon samples and data analysis

#### RNA isolation and purification

Total RNA was isolated and purified using TRIzol reagent (Invitrogen, Carlsbad, CA, USA) following the manufacturer’s procedure. The amount and purity of each RNA sample were quantified using a NanoDrop ND-1000 (NanoDrop, Wilmington, DE, USA).

#### cDNA library construction and bioinformatics analysis

The cDNA library was constructed, and we performed 150 bp paired-end sequencing on an Illumina NovaSeq 6000 (LC Bio, Hangzhou, China). Gene expression levels were estimated by calculating the value of fragments per kilobase of exon per million reads mapped (FPKM). We selected genes with a fold change > 2 and *P*-value < 0.05. Then, we analysed the enrichment of differentially expressed genes using GO enrichment analysis and the Kyoto Encyclopedia of Genes and Genomes (KEGG) pathway analysis.

### Characterisation and quantification of genes and proteins related to the immune response and intestinal barrier

#### Immunohistochemistry

Colon tissue pieces embedded in wax were sliced and dried. The sections were dewaxed in xylene and rehydrated in a gradient of alcohol to distilled water. Then, the sections were placed in antigen retrieval solution and heated for 10 min under low heat. After cooling, the samples were washed with 2% PBST and incubated with a blocking solution for 30 min. Next, the sections were incubated with primary antibody overnight, followed by incubation with secondary antibody overnight. After washing, the sections were incubated with ABC reagents for 30 min. The DAB chromogenic reagent was dripped onto the slice until the colour deepened to the correct hue. The sections were counterstained with haematoxylin, dehydrated and sealed. Finally, the sections were observed using a microscope, and images were acquired at 200× magnification. IHC quantification was performed using a parameter called the mean optical density detected using Image-Pro Plus (version 6.0, Rockville, MD, USA). This parameter was obtained by dividing the cumulative optical density value of each point on the picture by the area of the target distribution.

#### Quantitative RT-PCR analysis of gene expression

Briefly, total RNA was isolated using TRIzol reagent (Takara Biomedical Technology Co., Ltd., Beijing, China) and then reverse transcribed into cDNA using the PrimeScript™ RT reagent Kit and gDNA Eraser Kit (Takara Biomedical Technology, Beijing, China) following the manufacturer’s protocol. Next, mRNA levels were detected by standard real-time polymerase chain reaction (RT-PCR). RT-PCR was performed using a 7500 real-time PCR system (Applied Biosystems) with the SYBR Green mix kit (Takara Biomedical Technology, Beijing, China) to measure mRNA expression, with β-actin as a reference gene. Relative quantification of gene amplification by RT-PCR was performed using cycle threshold (CT) values. The relative mRNA expression of the selected genes was normalised to the control gene β-actin and determined using the 2^−ΔΔCt^ method [19]. All primer sequences are listed in Supplementary Table S1.

#### Enzyme-linked immunosorbent assay

The cytokines in colon homogenate and TJ proteins in mucosal tissue were analysed using an enzyme-linked immunosorbent assay (ELISA) kit (Hnybio, Shanghai, China) according to the manufacturer’s instructions.

### Statistical analysis

Phenotypic clinical evaluation, colonic SCFA concentration and immune cell and key immune factor content data were analysed using SPSS statistical software with the *t*-test. The results are presented as the mean and standard error of the mean (SEM). Statistical significance was based on a *P*-value < 0.05 (*P < 0.05, **P < 0.01 and ***P < 0.001). Microbial families and genera were compared using linear discriminant analysis effect size (LEfSe), and significant differences were denoted by an LDA score > 2 and *P*-value < 0.05. For metabolome data, partial least squares discriminant analysis (PLS-DA) and *t*-tests were performed between control and diseased individuals of the two breeds, with *P* < 0.05 and *VIP* > 1 indicating significantly different metabolites.

## Results

### Pig breeds exhibit varying degrees of phenotypic characteristics of disease resistance

**Fig. 1:**
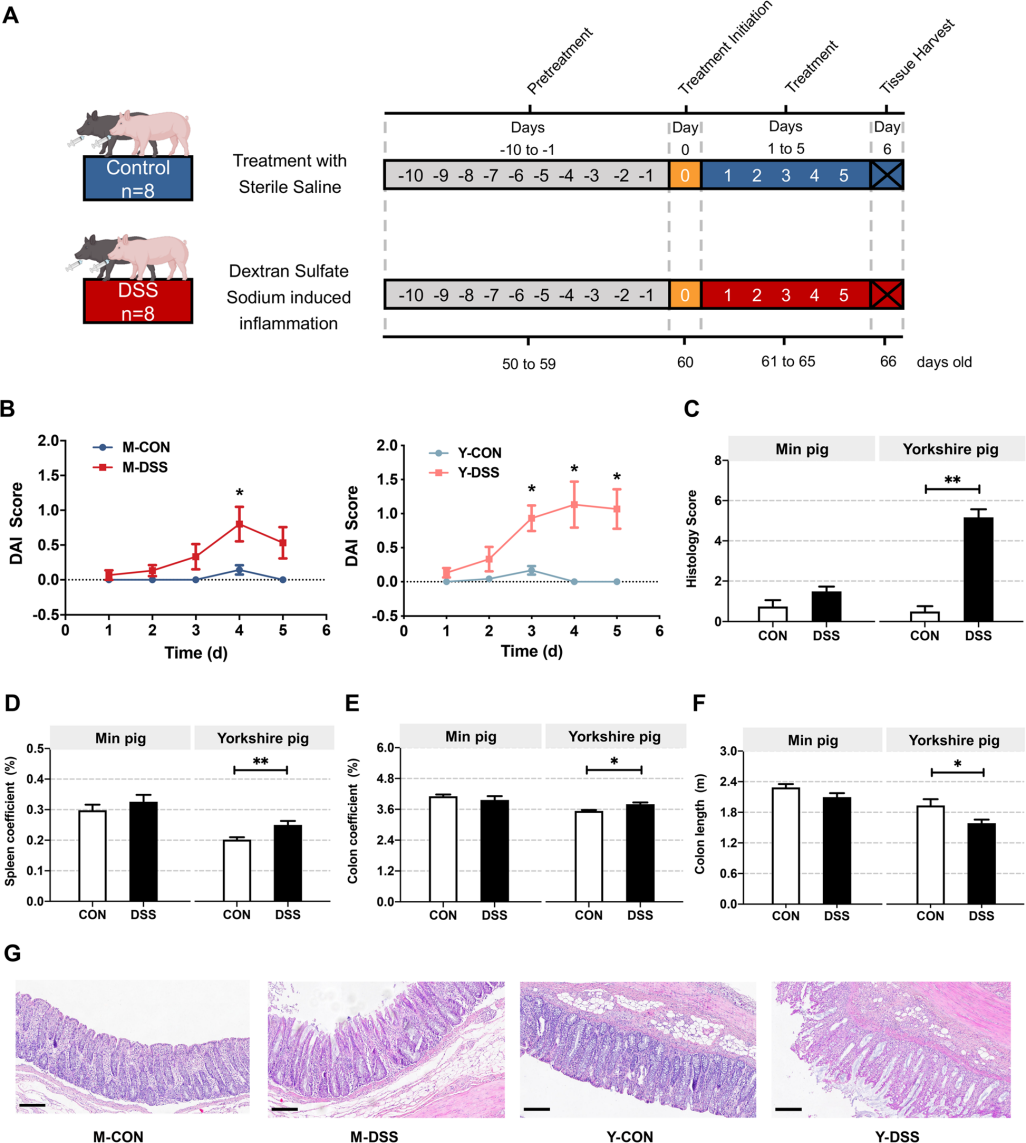
Response of Min pigs and Yorkshire pigs to DSS-induced colitis. **a** Min pigs and Yorkshire pigs were administered CON (control, sterile saline) or DSS (4% DSS in 100 mL of water) via gavage for 5 days. The first dose had a volume of 200 mL. **b** Plot of the disease activity index (DAI) values of Min pigs and Yorkshire pigs against the number of days (*n* = 8 samples/group). DAI values were calculated as (weight loss rate score + stool consistency score + bloody stool score)/3. **c** Histological activity index (HAI) values of Min pigs and Yorkshire pigs after DSS treatment. Comparison of **d** spleen coefficient, **e** colon coefficient and **f** colon length in Min pigs and Yorkshire pigs after DSS treatment (*n* = 8 samples/group). **g** Distal colon inflammation and histopathology score of Min pigs and Yorkshire pigs after DSS treatment (*n* = 5 samples/group). Scale bar, 200 μm. Data are presented as the mean ± SEM and were analysed by the *t*-test; significance is reported as *P < 0.05, **P < 0.01

The unambiguously different disease resistance phenotypes of Min and Yorkshire pigs may be related to gut microbiota composition and host gut biological function. For this reason, it is imperative to first determine if there are distinct phenotypic differences between Min pigs and Yorkshire pigs. Therefore, we compared control Min pigs with acute colitis models of Min pigs (M-CON vs. M-DSS) and control Yorkshire pigs with acute colitis models of Yorkshire pigs (Y-CON vs. Y-DSS) (Fig. 1a). To enable a more intuitive comparison of two pig breeds, the disease activity index (DAI) and histological activity index (HAI) evaluation were used to compare disease activity between control individuals and DSS-induced colitis models. The DAI and HAI were increased in M-DSS and Y-DSS pigs compared with M-CON and Y-CON pigs, confirming the successful establishment of experimental colitis. Although impairments were observed in both M-DSS and Y-DSS pigs, Y-DSS pigs exhibited earlier and more severe organismal damage, as indicated by high body weight loss, stool consistency and haematochezia scores and extensive mucosal disease and crypt loss (*P* < 0.01) (Fig. 1b, c and g). These observations suggested differences in disease resistance between the two pig breeds. An important element of immunophenotype is the proportion of the colon and spleen, an immune organ containing abundant lymphocytes, in overall body weight. Significant spleen and colon enlargement were observed only in Y-DSS pigs, in addition to significantly shorter colon length (*P* < 0.05), indicating severe inflammation (Fig. 1d–f).

### Colonic microbiota and metabolites undergo dramatic remodelling between control and diseased individuals in both two pig breeds

**Fig. 2:**
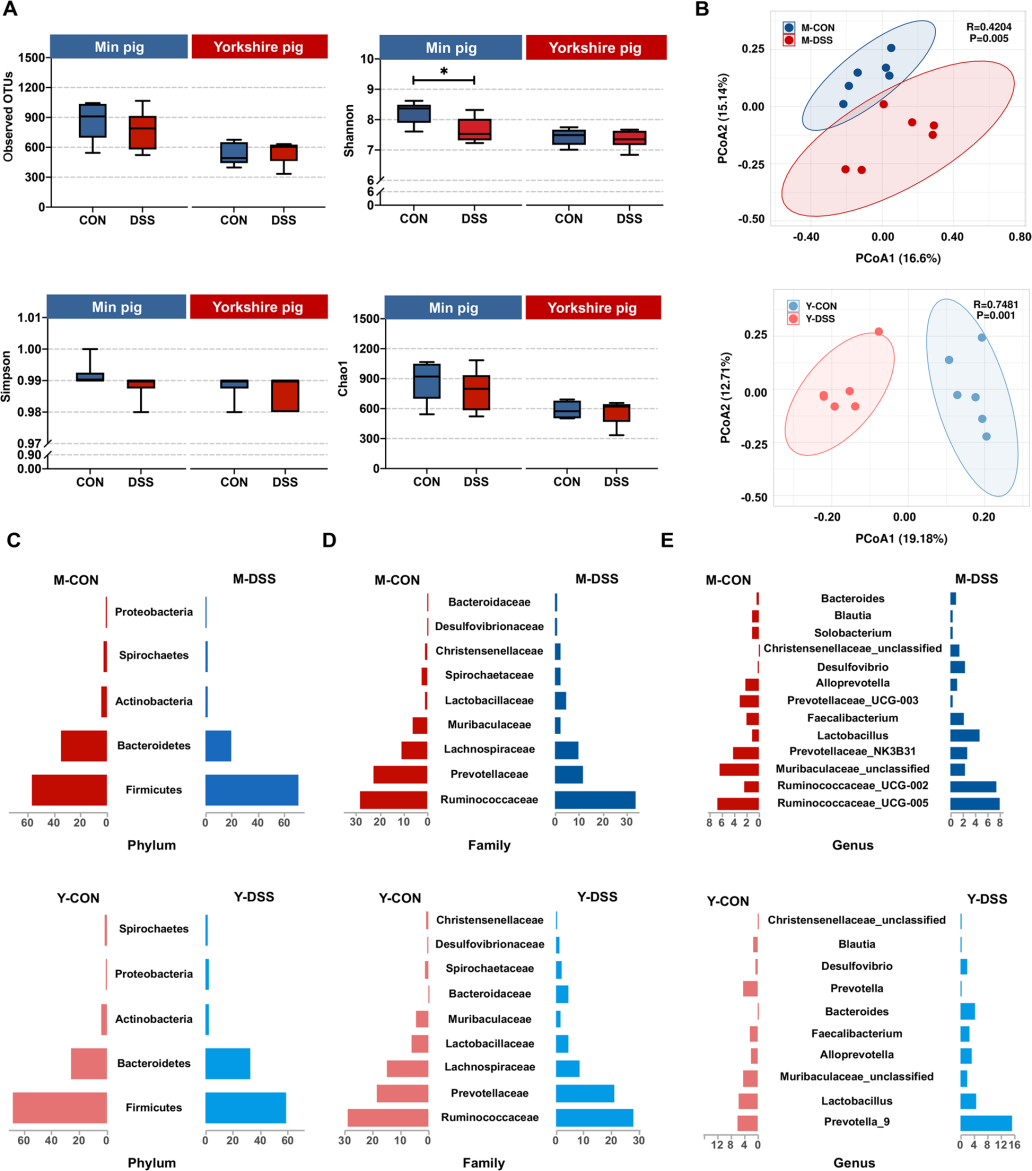
Shifts in the colonic microbiota composition of Min pigs and Yorkshire pigs after DSS treatment. **a** Comparison of the observed OTUs and Shannon, Simpson and Chao1 indices of the gut microbiota between control and diseased Min pigs and Yorkshire pigs (*n* = 6 samples/group). **b** Principal coordinate analysis (PCoA) plot of the microbial compositional profiles between control and diseased Min pigs and Yorkshire pigs (*n* = 6 samples/group). Relative abundance of the colonic microbiota at the **c** phylum, **d** family and **e** genus levels in Min pigs and Yorkshire pigs after DSS treatment (*n* = 6 samples/group)

Intrigued by the results of the phenotypic characterisation, we next sought to determine the bacterial composition of the microbiota in control and diseased individuals of the two pig breeds to elucidate any compositional differences. We calculated α-diversity using the richness and Shannon indices as a measure of species diversity across the samples. α-diversity did not differ between Y-CON and Y-DSS pigs but was slightly lower (although still high) in M-DSS pigs than in M-CON pigs (Fig. 2a). Compared with control pigs, pigs with DSS-induced colitis showed significant differences in β-diversity, a measure of compositional similarity, as measured by unweighted UniFrac distances. It is known that pigs with DSS-induced colitis could alter the gut microbiota, and our findings further supported significant changes in bacterial composition in M-DSS and Y-DSS pigs (Fig. 2b). To further understand the microbial dissimilarities between control and diseased individuals of the two breeds, we evaluated the taxonomic composition of the colonic microbiota. The clusters of samples that significantly separated the microbiota according to breed and treatment are shown in Fig. 2c, d and e and Supplementary Fig. 1. M-CON pigs had a diverse community of different bacterial phyla in the colon, whereas M-DSS pigs displayed an increase in *Firmicutes* and major reductions in *Bacteroidetes* and *Spirochaetes* (Fig. 2c). A detailed investigation of the families belonging to *Firmicutes* revealed increases in *Ruminococcaceae*, *Christensenellaceae* and *Lactobacillaceae* in the colons of M-DSS pigs. Among families belonging to *Bacteroidetes*, *Prevotellaceae* and *Muribaculaceae* decreased, while *Bacteroidaceae* increased. Different results were obtained for Yorkshire pigs (Fig. 2d). At the phylum level, Y-DSS pigs displayed a reduction in *Firmicutes* and increases in *Bacteroidetes*, *Proteobacteria* and *Spirochaetes* compared with Y-CON pigs (Fig. 2c). The decreased abundance of *Firmicutes* included a wide range of families, including *Lachnospiraceae*, *Christensenellaceae* and *Lactobacillaceae*, and the increased abundance of *Bacteroidetes* was represented by increases in the families *Prevotellaceae* and *Bacteroidaceae* (Fig. 2d)*.*

**Fig. 3:**
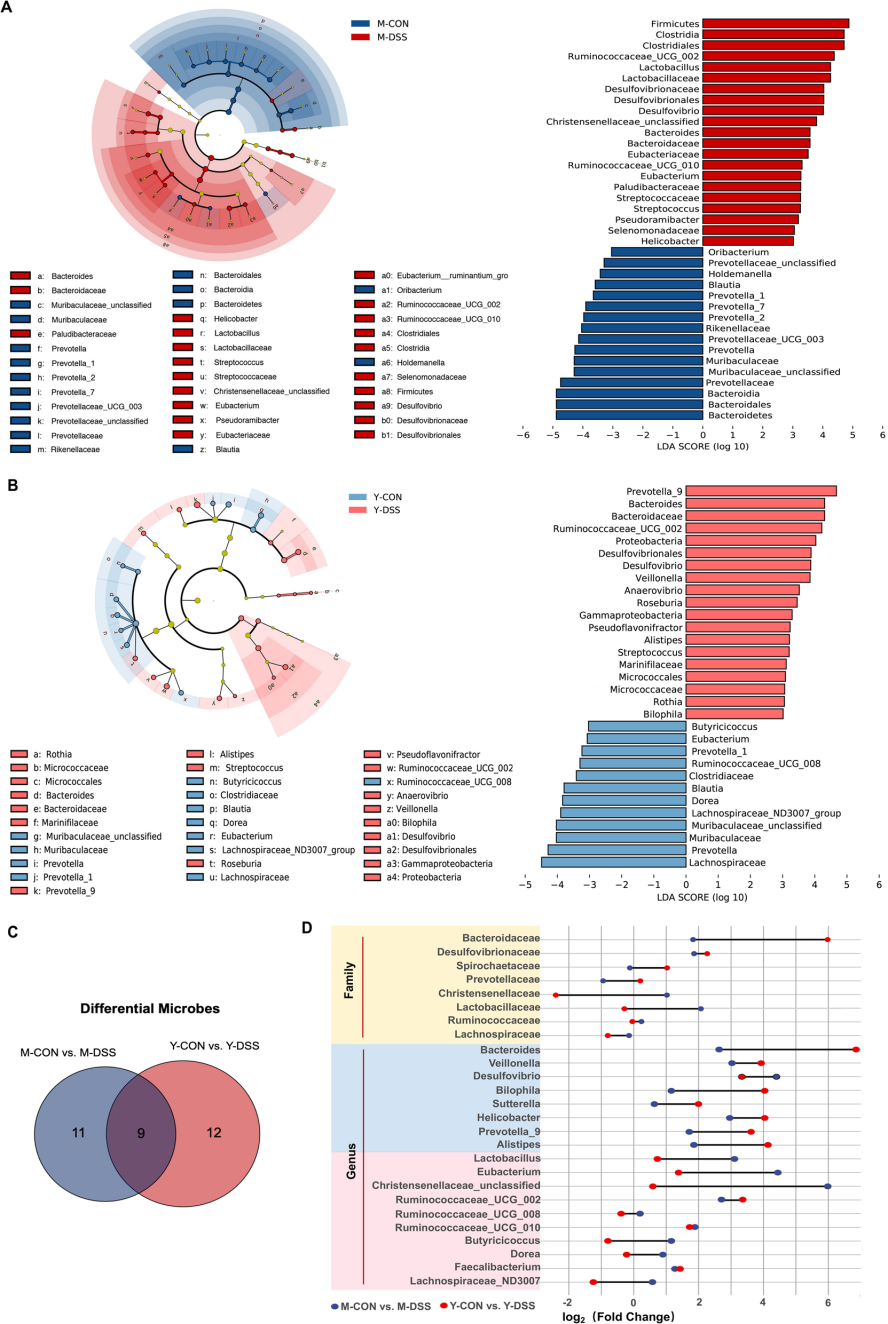
Differences in microbial abundance between control and diseased Min pigs and Yorkshire pigs. Cladogram. LDA distribution. Linear discriminate analysis effect size (LEfSe) was used to analyse the differences in microbial abundance in **a** Min pigs and **b** Yorkshire pigs after DSS treatment (*n* = 6 samples/group). **c** Venn diagram for differential microbes in the comparisons M-CON vs. M-DSS and Y-CON vs. Y-DSS (*n* = 6). **d** Fold changes of differential microbes in the comparisons M-CON vs. M-DSS and Y-CON vs. Y-DSS (*n* = 6)

To identify specific bacterial genera that were characteristic of the two breeds, linear discriminant analysis effect size (LEFSe) was used to evaluate further the differences in bacterial composition between animals (Fig. 3a and b). In our study, differential abundance tests identified similarities in the dysbiosis patterns of the Min pigs and Yorkshire pigs. Our data indicated that *Bacteroides*, *Desulfovibrio* and *Streptococcus*, which were associated with intestinal inflammation [20, 21], were enriched in diseased individuals of two breeds, while *Muribaculaceae* and *Blautia* decreased in diseased individuals of two breeds (Fig. 3c and d). Another critical characteristic of dysbiosis in Yorkshire pigs was that diseased individuals demonstrated enrichment of *Gammaproteobacteria*, *Bilophila*, *Veillonella* and *Prevotellaceae* genera and depletion of *Eubacterium*, *Dorea*, *Butyricicoccus* and *Lachnospiraceae*. Of interest, many potentially beneficial microbes, such as *Ruminococcaceae_UCG-02*, *Ruminococcaceae_UCG-10*, *Lactobacillus*, *Clostridia*, *Eubacterium* and unclassified *Christensenellaceae*, were increased in M-DSS pigs (Fig. 3d) but not Y-DSS pigs.

**Fig. 4:**
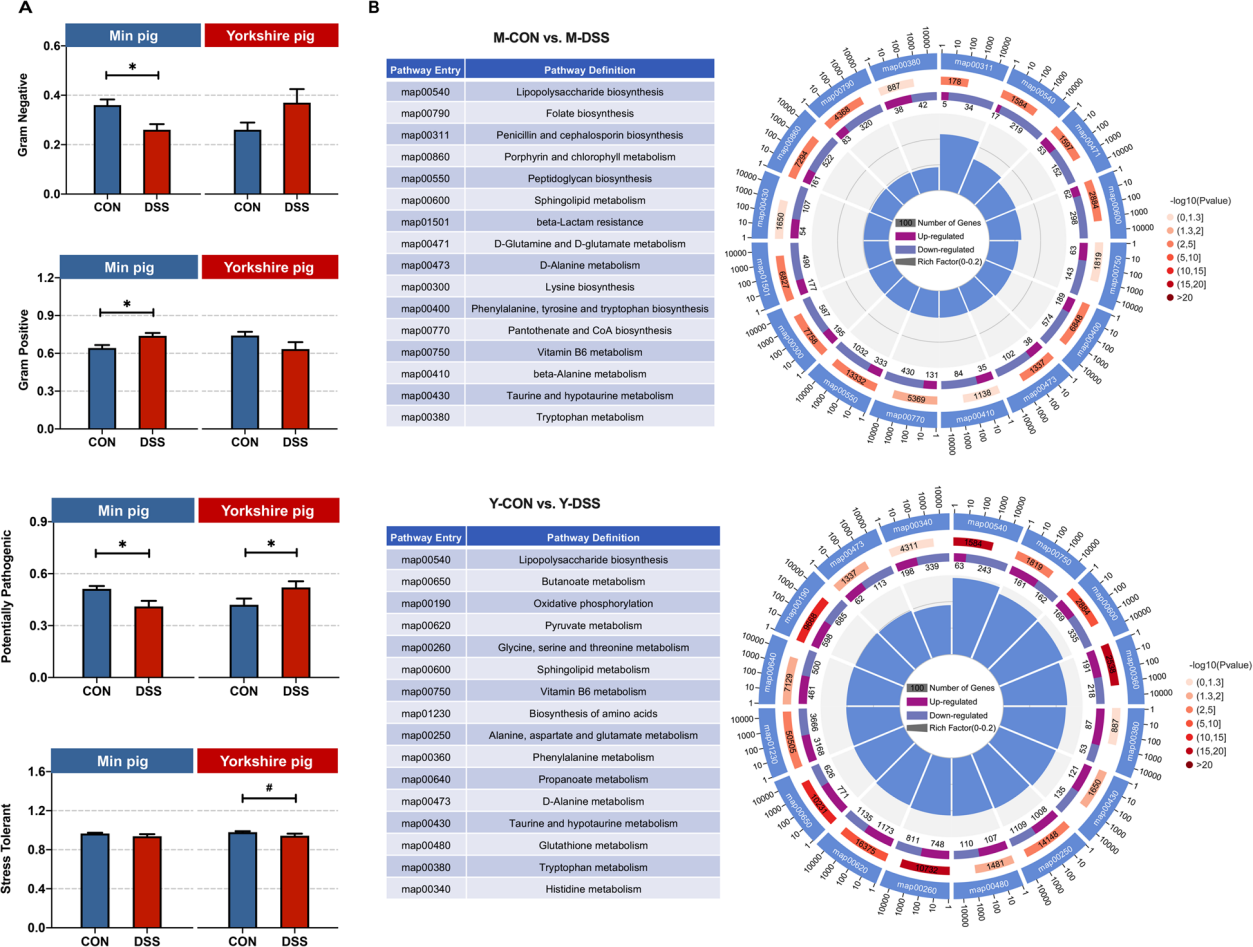
Differential functions of the microbiome between control and diseased Min pigs and Yorkshire pigs. **a** Bacterial community phenotypes of control and diseased Min pigs and Yorkshire pigs were predicted using BugBase (*n* = 6 samples/group). **b** Comparison of microbial KEGG modules between M-CON and M-DSS pigs and Y-CON and Y-DSS pigs (*n* = 6)

The data from the two breeds indicated that commensal bacteria of Min pigs might play important roles in the resistance to acute colitis. The phenotype prediction results based on the bacterial communities closely matched our experimental findings (Fig. 4a). Gram-negative bacteria were the most common co-pathogens [22]. In M-DSS pigs, the drastic decrease in the abundance of gram-negative bacteria was accompanied by an increase in gram-positive bacteria (*P* < 0.05), suggesting that potential pathogenicity decreased. In addition, the prediction results also revealed that potential pathogenicity decreased in M-DSS pigs but increased in Y-DSS pigs. The functions of the microbiome were determined by evaluating the Kyoto Encyclopedia of Genes and Genomes (KEGG) profiles (Fig. 4b). In the KEGG profiles, several third-level categories were considered microbial metabolic pathways, with “lipopolysaccharide biosynthesis”, “sphingolipid metabolism”, “beta-alanine metabolism”, “taurine and hypotaurine metabolism” and “tryptophan metabolism” being the most abundant in the two breeds. Some pathways, such as “butanoate metabolism”, were enriched in Yorkshire pigs.

**Fig. 5:**
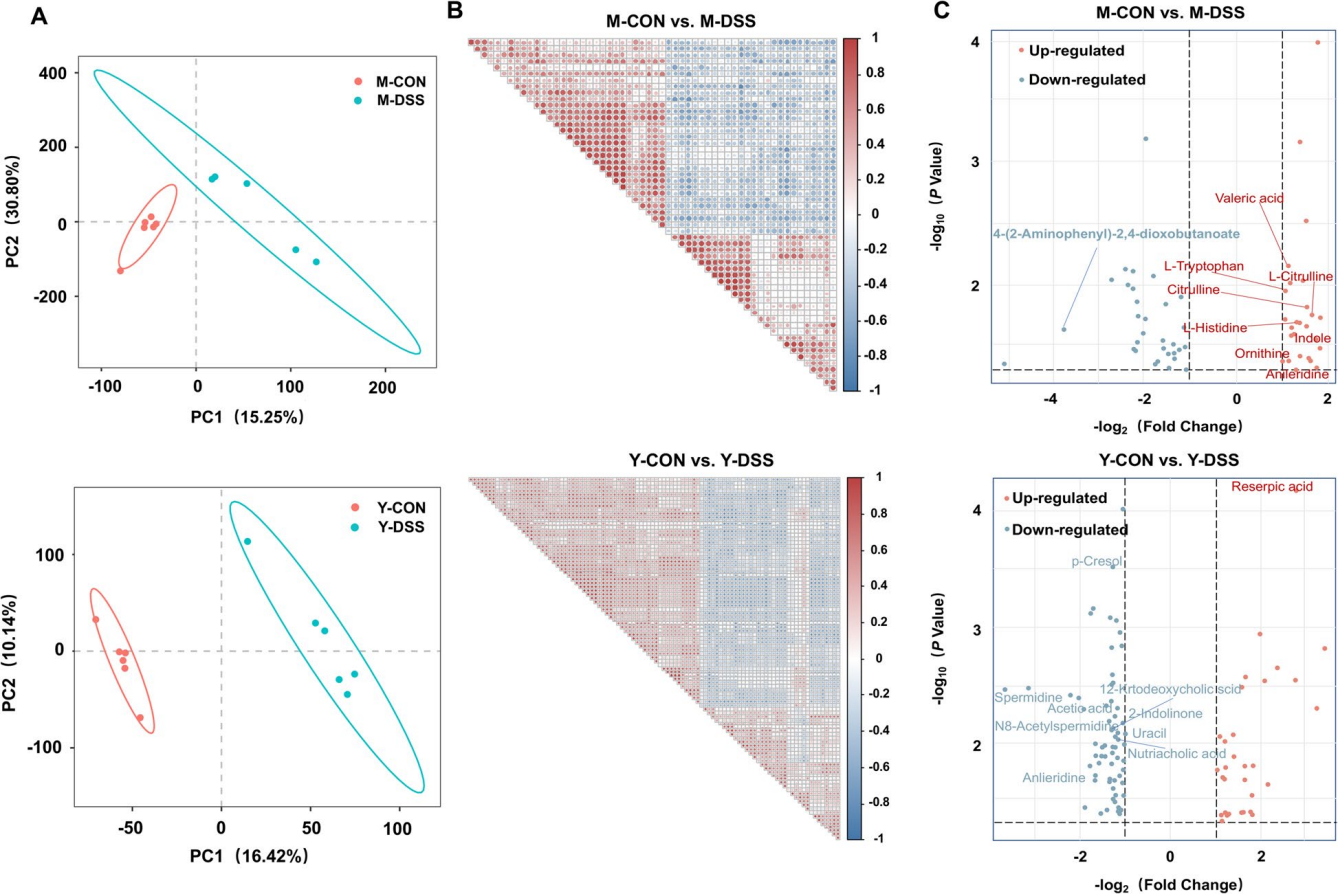
Colon metabolome changes. **a** Partial least squares discriminant analysis (PLS-DA) of metabolite composition in Min pigs and Yorkshire pigs after DSS treatment (*n* = 6 samples/group). **b** Correlations of the metabolites between control and diseased Min pigs and Yorkshire pigs (*n* = 6 samples/group). **c** Changes in colonic metabolites in Min pigs and Yorkshire pigs after DSS treatment (*n* = 6 samples/group)

To gain further insight into the metabolic changes in the two breeds after DSS treatment, untargeted metabolomics analysis of colon content was performed. A total of 1578 compounds were identified in the colon metabolome. Precise results were obtained with partial least squares discriminant analysis (PLS-DA), which indicated a significant change in colonic metabolites after DSS treatment (Fig. 5a). The metabolites in each comparison were correlated with each other (Fig. 5b). Univariate analysis of fold change, *t*-test and variable importance in projection (VIP) filtering of the expressed metabolites indicated that 51 metabolites were significantly different in Min pigs, including 26 that were significantly higher and 25 that were relatively low in M-DSS pigs (Fig. 5c and Supplementary Fig. 2). A total of 93 metabolites were significantly different in Y-DSS pigs compared with Y-CON pigs, including 31 upregulated and 62 downregulated metabolites (Fig. 5c).

**Fig. 6:**
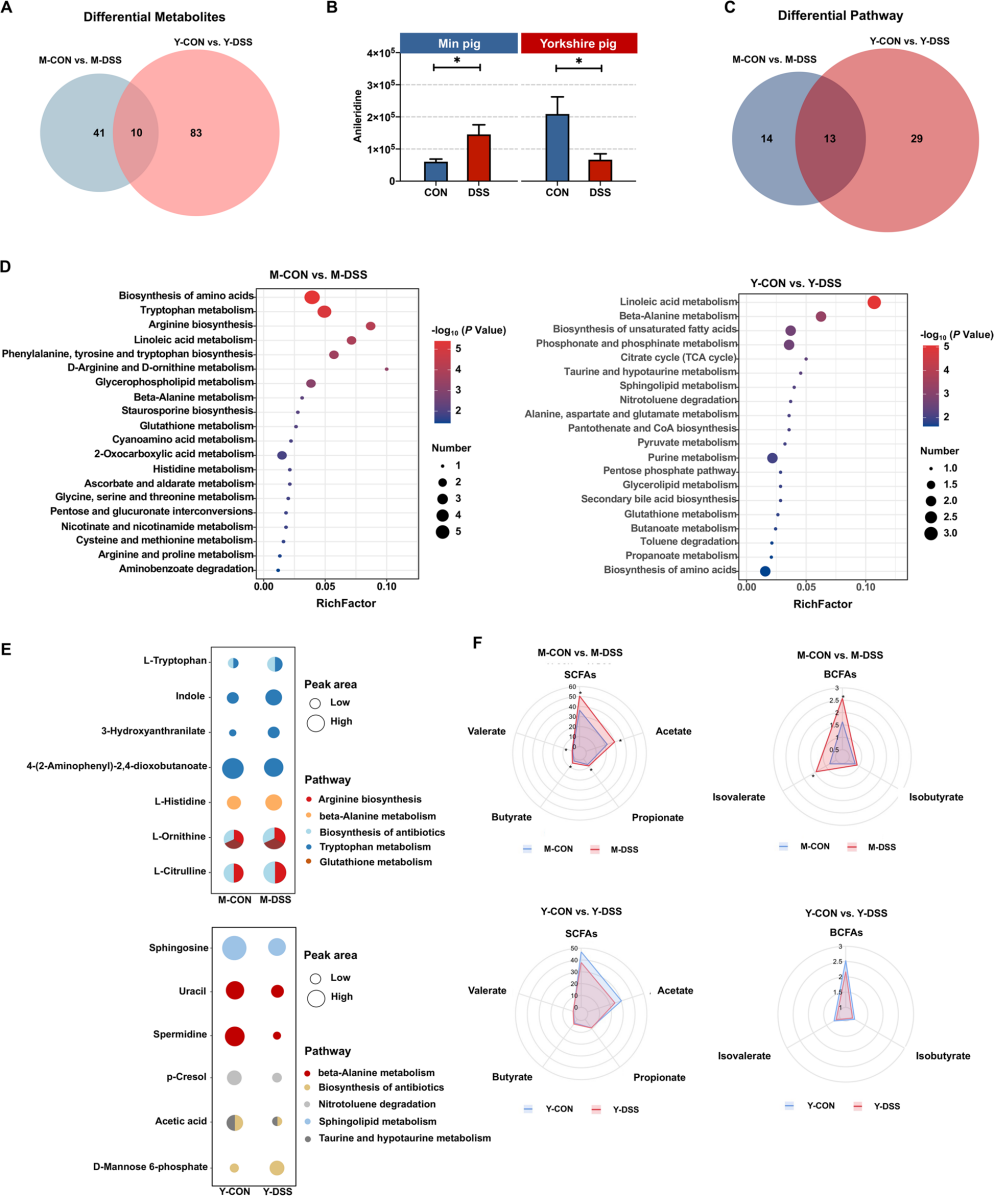
Comparison of colonic metabolites in control and diseased Min pigs and Yorkshire pigs. **a** Venn diagram for differential metabolites in the comparisons M-CON vs. M-DSS and Y-CON vs. Y-DSS. **b** Common differential metabolites between control and diseased Min pigs and Yorkshire pigs. **c** Venn diagram for differential pathways of the comparisons M-CON vs. M-DSS and Y-CON vs. Y-DSS. **d** Pathway enrichment analysis was performed using the significantly different metabolites between control and diseased Min pigs and Yorkshire pigs (*n* = 6 samples/group). **e** The major pathways of significantly different metabolites in Min pigs and Yorkshire pigs after DSS treatment. **f** Concentrations of short-chain fatty acids (SCFAs) in Min pigs and Yorkshire pigs after DSS treatment (*n* = 8 samples/group)

Interestingly, the Venn diagram revealed 10 differential metabolites in common between the two comparisons (M-CON vs. M-DSS, Y-CON vs. Y-DSS) (Fig. 6a). Nine of the common differential metabolites showed the same trends between control and diseased individuals of the two breeds; the remaining metabolite, anileridine, showed opposing directions. Anileridine was significantly higher in M-DSS pigs but significantly lower in Y-DSS pigs than in the corresponding controls (Fig. 6b). To determine the functions of the altered metabolites, we performed KEGG enrichment analysis (Fig. 6c and d), and the results were in basic agreement with the microbiome functions. The common pathways enriched in comparisons of M-CON vs. M-DSS and Y-CON vs. Y-DSS included linoleic acid metabolism, biosynthesis of amino acids, glutathione metabolism and beta-alanine metabolism. Notably, the significantly altered metabolites related to beta-alanine metabolism exhibited opposing trends of change between the two breeds. These metabolites increased in M-DSS pigs, indicating activation of this pathway but decreased in Y-DSS pigs. The specific pathways enriched in M-DSS pigs compared with M-CON pigs were arginine biosynthesis, tryptophan metabolism and biosynthesis of antibiotics. Specifically, we found that some intermediate metabolites involved in the biosynthesis of antibiotics, such as ornithine, L-citrulline and L-tryptophan, were significantly increased in M-DSS pigs. All significantly altered metabolites involved in tryptophan metabolism were upregulated in M-DSS pigs (Fig. 6e), suggesting that the metabolome of Min pigs may undergo a superior adaptive adjustment by increasing the accessibility of tryptophan to the host. The specific pathways enriched in Y-DSS pigs compared with Y-CON pigs were sphingolipid metabolism, nitrotoluene degradation and taurine and hypotaurine metabolism. The major intermediate metabolites involved in these pathways, such as spermidine, sphingosine and acetic acid, were downregulated in Y-DSS pigs compared with Y-CON pigs (Fig. 6e). In addition to the relative concentrations of colonic small molecules that were identified by metabolomics, the absolute concentrations of the total short-chain fatty acids (SCFAs), acetate, propionate, butyrate, valerate, isobutyrate and isovalerate were quantified (Fig. 6f). Acetate, propionate, butyrate, valerate and isovalerate were significantly higher in M-DSS pigs than in M-CON pigs (*P* < 0.05) but lower in Y-DSS pigs than in Y-CON pigs, potentially indicating a relatively lower self-adjustment capacity of Yorkshire pigs during acute colitis.

Spearman’s correlation coefficient (*R* > 0.50, *P* < 0.05) assessed the correlation between the colonic microbiota and metabolites. In Min pigs (Supplementary Fig. 3a), positive correlations were observed mainly between potentially beneficial microbes *Lactobacillus*, *Eubacterium*, *Blautia*, *Ruminococcaceae_UCG_002* and *Christensenellaceae* and acetate, L-tryptophan, butyrate, benzoic acid and anileridine (0.58 < *R* < 0.93, *P* < 0.05). These microbiota and metabolites are involved in the biosynthesis of amino acids, biosynthesis of antibiotics, linoleic acid metabolism, glycerophospholipid metabolism and tryptophan metabolism. The Spearman’s rank correlation network showed that potentially beneficial microbes might assist in metabolite recovery and support normal gut function (Supplementary Fig. 3c). In Yorkshire pigs (Supplementary Fig. 3b), negative correlations mainly existed between potentially harmful microbes *Veillonella*, *Alistipes*, *Bacteroides*, *Bilophila* and *Desulfovibrio* and fatty acyls, phenols, imidazopyridine, isoflavonoids, sterol lipids, carboxylic acids and derivatives (0.58 < *R* < 0.96, *P* < 0.05), which are involved in the biosynthesis of antibiotics, taurine and hypotaurine metabolism, butanoate metabolism, linoleic acid metabolism, secondary bile acid biosynthesis and beta-alanine metabolism. The correlation network was built from the Spearman’s nonparametric rank correlation coefficient between microbiota and the above metabotypes (Supplementary Fig. 3d). Potentially harmful microbes were negatively correlated with most of the above metabotypes, including metabolites such as N8-acetylspermidine, spermidine, 12-ketodeoxycholic acid, nutriacholic acid, reserpic acid, kanzonol and valerate. These data suggested that the changes in microbes in Yorkshire pigs were associated with metabolic dyshomeostasis and interfered with normal intestinal function.

### Gut barrier function responds differently to DSS treatment between Min and Yorkshire pigs

**Fig. 7:**
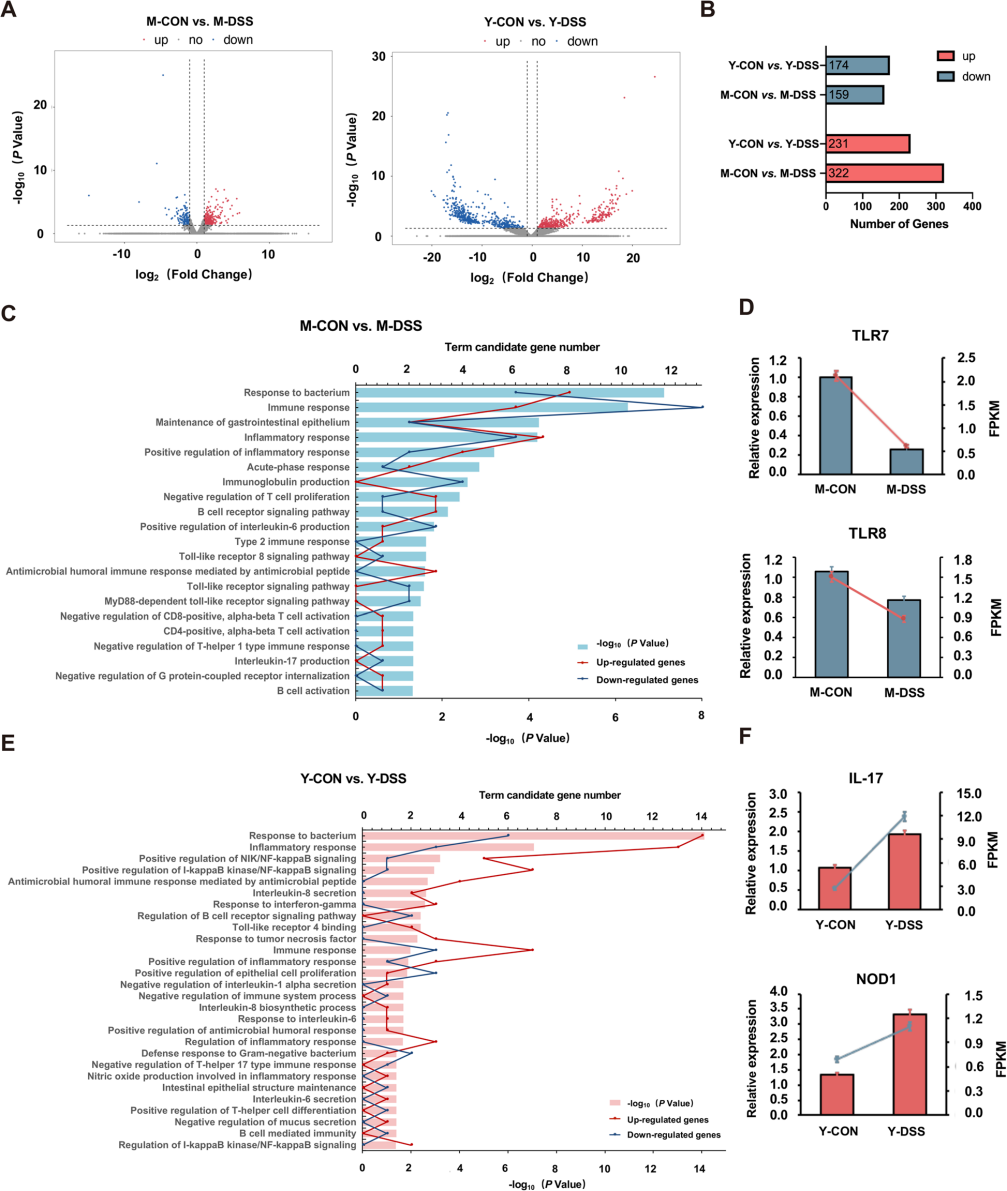
RNA-seq analysis of the colon of Min pigs and Yorkshire pigs. **a** Volcano plot of differentially expressed genes between control and diseased Min pigs and Yorkshire pigs. **b** Numbers of upregulated and downregulated differentially expressed genes in Min pigs and Yorkshire pigs after DSS treatment. GO functional enrichment analysis of differentially expressed genes and qRT–PCR validation of the biological process of immunity and intestinal structure in **c**–**d** Min pigs and **e**–**f** Yorkshire pigs after DSS treatment

Gut barrier function comprises three major parts: a mechanical barrier, an ecological barrier and an immunological barrier [23]. Disorganisation of the ecological barrier (microbial dysbiosis) often leads to dysfunction of the mechanical barrier and immunological barrier. To explore the underlying mechanisms of core bacterial protection of the host colon, the colon gene expression profiles of the two breeds were quantified by RNA-seq analysis. Figure 7a shows the volcano plots generated to visualise the distribution of expressed genes between control and diseased Min pigs and Yorkshire pigs. In total, 322 genes were upregulated and 159 genes were downregulated in M-DSS pigs compared with M-CON pigs, and 231 genes were upregulated and 174 genes were downregulated in Y-DSS pigs compared with Y-CON pigs (Fig. 7b). To gain insight into the biological processes of the immunological barrier and mechanical barrier that were differentially regulated between control and diseased Min and Yorkshire pigs, gene ontology (GO) enrichment analysis was performed (Fig. 7c–f). Compared with M-CON pigs, several terms involved in immunity, such as response to bacterium, immune response, inflammatory response, acute-phase response, type-2 immune response, CD4-positive, alpha-beta T-cell activation, negative regulation of T-helper 1 type immune response, negative regulation of CD8-positive, alpha-beta T-cell activation, antimicrobial humoral immune response mediated by antimicrobial peptide, toll-like receptor signalling pathway and MyD88-dependent toll-like receptor signalling pathway, were enriched in M-DSS pigs. The expression trends of the *TLR7* and *TLR8* genes identified by RNA-seq analysis were validated by qRT-PCR, which was downregulated in M-DSS pigs (*P* < 0.05) (Fig. 7d). In Y-DSS pigs, GO analysis showed that the following terms belonging to biological processes of immunity and the intestinal structure were enriched compared with Y-CON pigs: response to bacterium, inflammatory response, positive antimicrobial humoral immune response mediated by antimicrobial peptide, Toll-like receptor 4 binding and immune response. A few pathways were enriched in Y-DSS pigs but not M-DSS pigs, such as regulation of NIK/NF-κB signalling and positive regulation of I-κB kinase/NF-κB signalling. The genes in these pathways exhibited strong upregulation in Y-DSS pigs (*P* < 0.05), which indicated that inflammation was exacerbated in Yorkshire pigs. Some pathways involved in the immune response, including interleukin-8 secretion, interleukin-6 secretion, response to tumour necrosis factor, response to interferon-gamma and T-helper 17 type immune response, were also activated in Yorkshire pigs. These results indicated that the Th17-type immune response, an important driver of autoimmune disease with inflammatory properties, was dominant in Yorkshire pigs [24]. Matching the altered genes to the KEGG database indicated that the IL-17 signalling pathway was enriched in Yorkshire pigs (Supplementary Fig. 4).

**Fig. 8:**
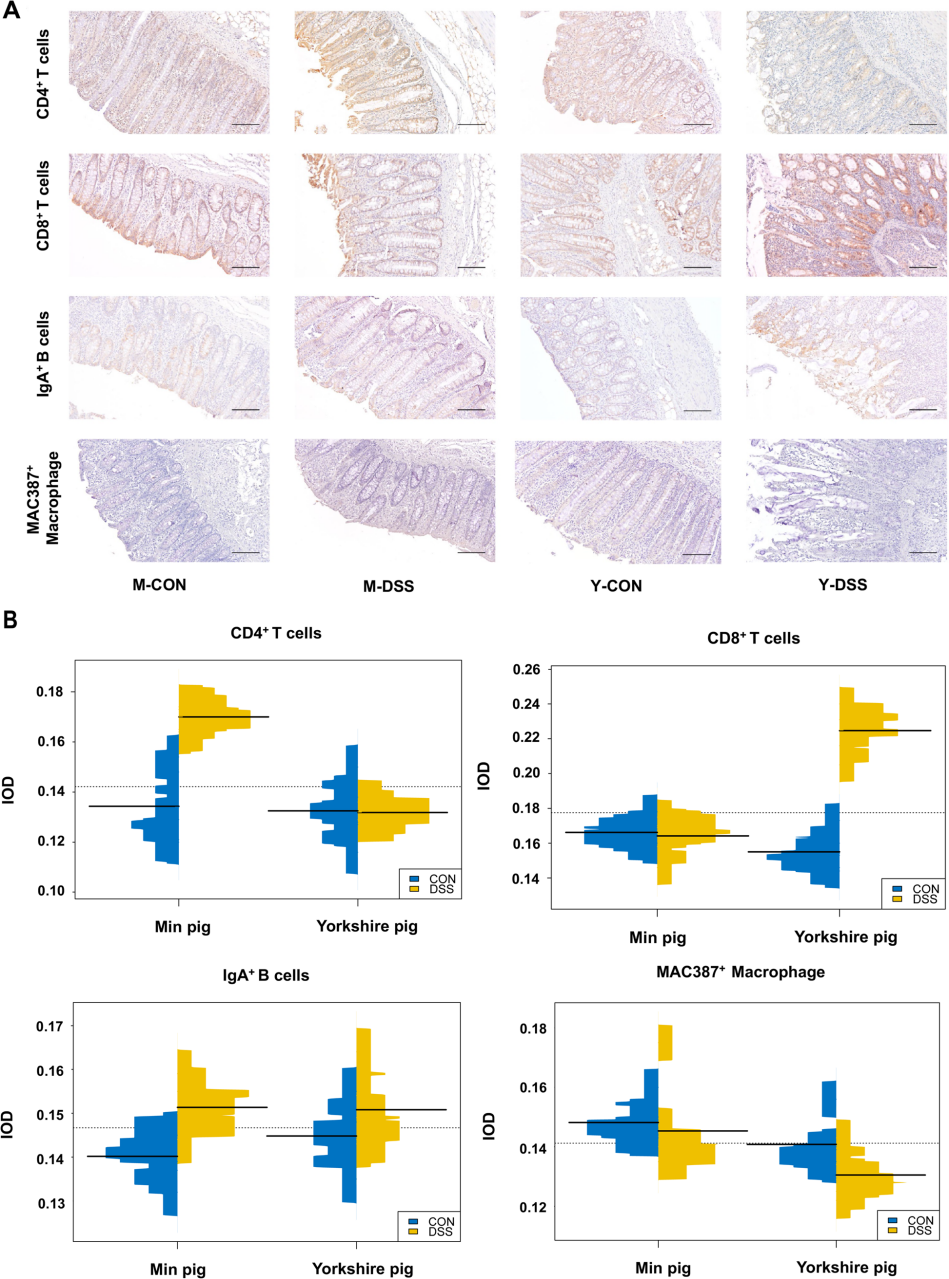
Changes in immune cells in the pig colon after DSS treatment. **a** Immunohistochemical staining (IHC) shows the numbers and distribution of CD4^+^ T cells, CD8^+^ T cells, IgA^+^ B cells and MAC387^+^ macrophages in control and diseased Min pigs and Yorkshire pigs (*n* = 4 samples/group). **b** Qualitative comparisons of the numbers of immune cells. The results are expressed as integrated optical density (IOD)

Since several terms involved in the mechanical barrier and immunological barrier were enriched in RNA-seq analysis, immune-related biological processes and pathways were analysed in the colon samples. We validated the activation of these biological processes and identified their consequences. For the immunological barrier, the most striking difference in immune cells was that CD4^+^ T cells were significantly higher in M-DSS pigs than in M-CON pigs (*P* < 0.05). Conversely, CD8^+^ T cells were significantly higher in Y-DSS pigs than in Y-CON pigs (*P* < 0.05) (Fig. 8a and b).

**Fig. 9:**
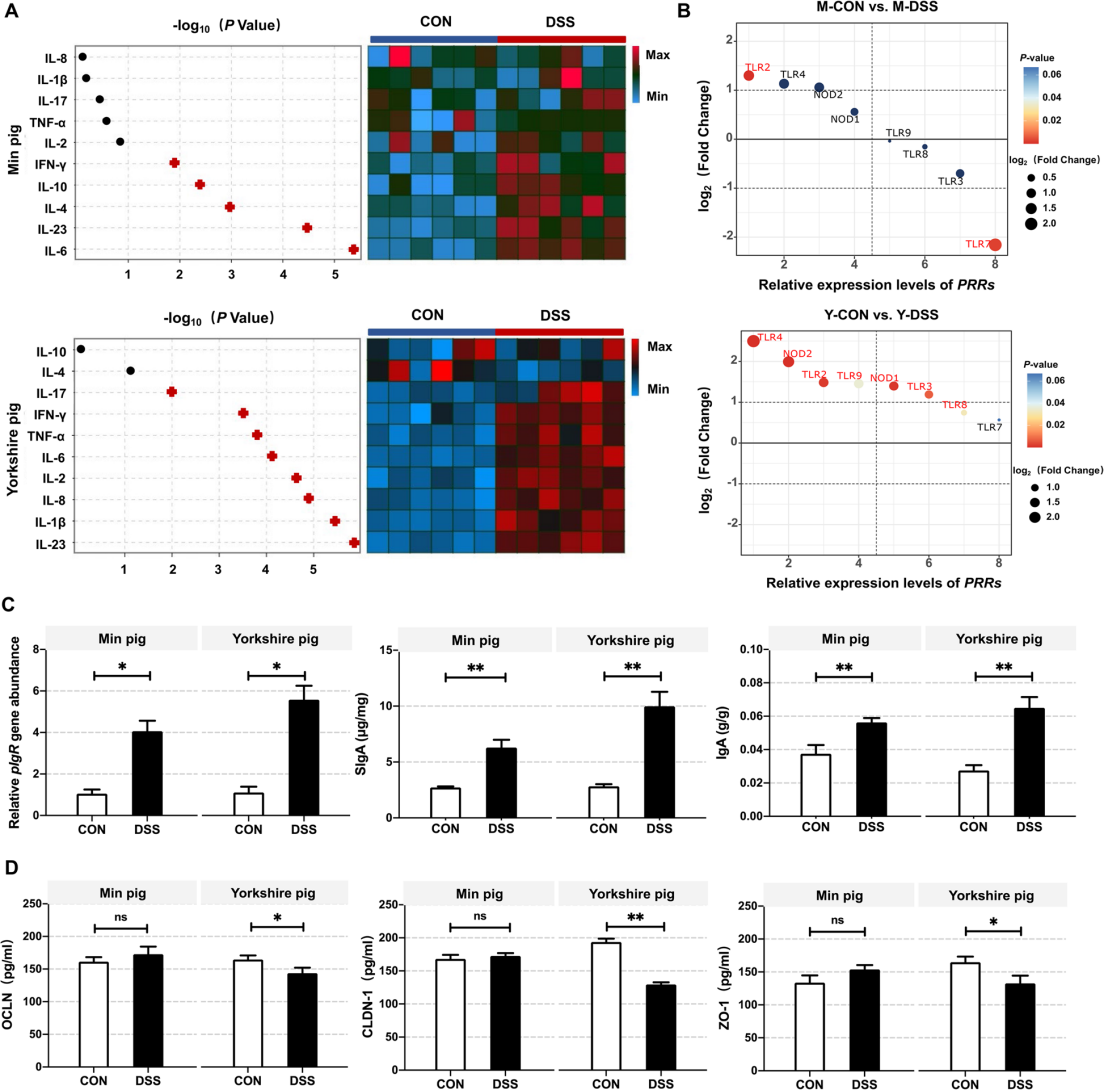
Analysis of the response of the gut barrier to inflammation based on transcriptome data. **a** Heat map showing the changes in inflammatory cytokines associated with different types of immunity in colon homogenate isolated from Min pigs and Yorkshire pigs after DSS treatment (*n* = 6 samples/group). **b** Relative expression levels of pattern recognition receptors (PRRs) in Min pigs and Yorkshire pigs after DSS treatment as detected by qRT-PCR (*n* = 6 samples/group). **c** Confirmation of autoantibody secretion in Min pigs and Yorkshire pigs after DSS treatment. **d** To evaluate the mechanical barrier, tight junction (TJ) proteins in Min pigs and Yorkshire pigs treated with DSS were quantified by ELISA

Among inflammatory cytokines, IL-4, IL-6, IL-10, IL-23 and IFN-γ levels were significantly increased in M-DSS pigs (*P* < 0.05). IL-4, IL-6 and IL-10 are the primary regulators of type-2 immune response [25, 26]. IL-1β, IL-2, IL-8, IL-17, IL-23, IFN-γ and TNF-α levels were increased in Y-DSS pigs compared with Y-CON pigs (*P* < 0.01) (Fig. 9a). Among PRR genes, *TLR2* was significantly downregulated in M-DSS pigs (*P* < 0.05), while *TLR7* was downregulated (*P* < 0.05). *TLR2*, *TLR3*, *TLR4*, *TLR8*, *NOD1* and *NOD2* were significantly upregulated in Y-DSS pigs (*P* < 0.05) (Fig. 9b). Both immunoglobulin A (IgA) and secreted IgA (SIgA) were increased in the colons of M-DSS and Y-DSS pigs (*P* < 0.05). Although polymeric immunoglobulin receptor (*pIgR*) levels were increased in M-DSS and Y-DSS pigs compared with the corresponding controls (*P* < 0.05), the increase was larger in Y-DSS pigs (Fig. 9c). With respect to the mechanical barrier, unlike Min pigs, tight junction (TJ) proteins such as occludin (OCLN), claudin (CLDN-1) and zonula occludens (ZO-1) were downregulated in Y-DSS pigs (*P* < 0.05) (Fig. 9d). Taken together, these results strongly suggest that Yorkshire pigs are more susceptible to inflammatory stimuli, leading to sustained inflammation.

## Discussion

Within the gut, the colon is home to the densest and most metabolically active community (comprising more than 10^13^ individual microbial cells) [27]. Although colitogenic pathobionts promote IBD development, commensal bacteria are also crucial for reducing IBD susceptibility [7]. The protective effect of commensal bacteria is evident from studies using germ-free mice, which are more susceptible to DSS-induced colitis than conventionally housed mice [28, 29]. The microbiota may have a major role in gut health, including the maturation of host immune responses, protection against enteric pathogen proliferation and response to or modification of specific drugs [6]. In the present study, diseased Min pigs exhibited mild organismal damage, as indicated by lower values of the body weight loss, stool consistency and haematochezia scores and limited mucosal disease and crypt loss, bolstering interest in whether changes in microbiota were associated with disease resistance. Important host-microbiota interactions in Min pigs are likely crucial for host physiology and the disease phenotype. We integrated microbiota sequencing, metabolomics and transcriptomics to investigate the host-microbiota crosstalk mechanisms that contribute to disease resistance.

In this study, all results for the acute colitis models were analysed separately for Min pigs and Yorkshire pigs. Progress to date indicates that IBD is a polymicrobial disease in which a combination of various gut microbial factors, abnormal immune responses and a weakened intestinal mucosal barrier leads to aberrant host-microbial interactions [30]. Intriguingly, the disease responses of the two breeds were not identical, although both exhibited inflammation. Consistent with many previous assessments of changes in microbiota in IBD [20, 31, 32], Y-DSS pigs exhibited a reduction in *Firmicutes* and increases in *Bacteroidetes*,* Proteobacteria* and *Spirochaetes* with Y-CON pigs. Surprisingly, however, similar changes were not observed in Min pigs. In contrast to Yorkshire pigs, M-DSS pigs displayed an increase in *Firmicutes* and major reductions in *Bacteroidetes* and *Spirochaetes*. At the genus level, more specific shifts were clearly observed. The higher incidence of populations of members of *Bacteroidetes* and *Proteobacteria* in the colonic mucosa of IBD patients has been reported [20, 21]. Our data demonstrated that *Bacteroides* and *Desulfovibrio*, as members of *Bacteroidetes* and *Proteobacteria*, respectively, were enriched in diseased individuals of the two breeds. Still, the extent and severity of these changes were less pronounced in Min pigs than in Yorkshire pigs. Increases in these bacteria are a frequent finding in IBD-associated dysbiosis, suggesting that these bacteria might function as potentially harmful microbes and play roles in the pathogenesis of the disease. A possible mechanism by which members of *Bacteroidetes* and *Prevotella* influence the severity of colitis is by removing sulphate from mucin with sulfatases, resulting in mucin degradation [33–35]. In addition, *Desulfovibrio*, as a sulphate-reducing bacteria (SRB), increases the concentration of hydrogen sulphide, eroding the intestinal epithelium [36]. Therefore, dysbiosis of intestinal microbiota might contribute to inflammation by an impairment of intestinal barrier. In our study, downregulated OCLN, CLDN-1 and ZO-1 levels were observed in Y-DSS pigs, which were consistent with our inference of intestinal barrier alteration with the role of microbes. Thus, it seems plausible to infer that more aggravative compromised intestinal barrier and more aggressive translocation of bacteria in Yorkshire pigs might influence each other, together exacerbating inflammation.

Additional key characteristics of dysbiosis in Y-DSS pigs were enrichment of *Gammaproteobacteria*, *Bilophila*, *Veillonella*, *Sutterella*, *Alistipes* and *Prevotella* and depletion of *Blautia*, *Eubacterium*, *Dorea*, *Butyricicoccus* and *Lachnospiraceae*. According to the Spearman’s rank correlation network, we found that the increases *in Bilophila*, *Alistipes* and *Bacteroidetes* in the colons of Yorkshire pigs were inversely correlated with the changes in acetate, deoxycholic acid and nutriacholic acid. The higher abundances of bile-tolerant microbes (*Bilophila*, *Alistipes* and *Bacteroidetes*) indicated that these bacteria might be the main contributors to the disruption of bile acid metabolism [37–39]. As supportive evidence, taurine and hypotaurine metabolism, involved in bile acid metabolism, were enriched both in KEGG profiles of microbiota and metabolite in Yorkshire pigs. Furthermore, members of *Blautia*, *Eubacterium*, *Dorea*, *Butyricicoccus* and *Lachnospiraceae* are among the main producers of SCFAs [40, 41]. SCFAs, particularly butyrate, have beneficial effects on the regulation of intestinal immune function and inhibition of intestinal inflammation [42, 43]. Depletion of these bacteria in Yorkshire pigs might be an influential factor to reduce SCFA concentrations and enlarge intestinal inflammation; these findings were reinforced by the results for inflammatory cytokine levels and mucosal barrier function. Remarkably, many potentially beneficial microbes, such as *Lactobacillus*, *Ruminococcaceae* genera, *Eubacterium* and *Christensenellaceae* genera belonging to *Firmicutes*, were increased in M-DSS pigs. The increased abundances of several *Ruminococcaceae* genera and *Eubacterium* could increase SCFA concentrations, thus protecting intestinal barrier function and exerting anti-inflammatory properties in the host [44]. Moreover, since *Christensenellaceae* is considered a potential probiotic that is negatively correlated with inflammation and metabolic-related diseases [45], the higher abundance of *Christensenellaceae* in M-DSS pigs suggests that defence against disease may be triggered in Min pigs. The more interesting finding, however, was the role of *Lactobacillus* in Min pigs. *Lactobacillus* expresses tryptophanase and is the most important commensal that metabolises tryptophan [46]. *Lactobacillus* protects the integrity of the intestinal epithelial barrier damaged by pathogenic bacteria and reduces the number of potentially pathogenic bacteria [47, 48]. Consistent with other studies, restoration of mechanical function and enrichment of tryptophan metabolites were observed in Min pigs. The data herein of microbiota and microbial pathways suggested that Min pigs can adjust the commensal bacteria to achieve disease remission.

The gut microbiota can produce diverse metabolites via anaerobic fermentation of exogenous undigested dietary components that reach the colon as well as endogenous compounds that are generated by microbes and the host. Microbial metabolites can access and interact with host cells to affect immune and inflammatory responses. Strong associations between colonic microbiota and metabolites were found by pairing metabolomics with microbiota sequencing analysis, showing changed levels of indole derivatives, bile acid, SCFAs and other metabolites. Previous studies have implicated that specific classes of metabolites, notably bile acids, SCFAs and tryptophan metabolites, were linked to the pathogenesis of IBD [37, 49]. We observed reduced SCFA concentrations and inhibition of taurine and hypotaurine metabolism in Yorkshire pigs. Min pigs exhibited a starkly different trend than Yorkshire pigs. In Min pigs, acetate, propionate, butyrate, valerate and isovalerate levels were enhanced, which were associated with the alteration of indigenous microbiota, and thus attenuated mucosal inflammation. In addition, tryptophan metabolism was promoted in Min pigs, which correspond to the increased abundances of *Lactobacillus* and other potentially beneficial microbes, indicating the gut microbiota can reduce tryptophan excretion and liberated more tryptophan to the host. Gut commensal bacteria can utilise tryptophan to ameliorate DSS-induced colitis by promoting colonic goblet cell differentiation and inducing mucin gene expression [37, 46]. These changes might explain the higher disease resistance of Min pigs compared with Yorkshire pigs. Additionally, the data on colonic metabolites that correlated negatively with disease activity showed that anileridine (spasmolytic action in the intestine [50]) and spermidine (protective role in colitis and colon carcinogenesis [51]) were decreased in Y-DSS pigs, suggesting an out-of-control state. Anileridine content was increased in M-DSS pigs, further supporting the ability of Min pigs to resist acute colitis. Overall, the results of the present study indicated that the beneficial gut microbes in Min pigs heal the colon and recover metabolites, given the good correlation between the gut microbiota and metabolites.

Microbial imbalance can directly contribute to gut barrier dysfunction and an immature immune system [52]. Gut barrier dysfunction is also strongly correlated with inflammation [53]. To further study the potential links of the gut microbiota with the host immune response and intestinal barrier function in the two pig breeds, RNA-seq analysis of the colons of Min pigs and Yorkshire pigs was performed. GO analyses showed enrichment of pathways involved in immune function, including the immune response, inflammatory response and antimicrobial humoral immune response mediated by antimicrobial peptides, in both Min and Yorkshire pigs, suggesting a response to exogenous stimuli. Although the responses were similar, the colonic inflammatory response was more active in Y-DSS pigs than in M-DSS pigs. Specifically, several PRRs signalling and downstream inflammation-related pathways, such as Toll-like receptor 4 binding, NOD-like receptor signalling, NIK/NF-κB, I-κB kinase/NF-κB signalling and proinflammatory cytokines secretion pathways, were enriched in Yorkshire pigs but not Min pigs. In agreement with the GO enrichment results, the increase of some NLR and TLR genes and their downstream proinflammatory factors were observed in Y-DSS pigs but not M-DSS pigs, which were confirmed by Elisa analysis and real-time PCR. It has been reported that PRRs, such as TLR4, NOD1 and NOD2, initiate inflammatory responses by sensing microbe-associated molecular patterns (MAMP) [54, 55]. PRRs serve the function of distinguishing potentially beneficial microbes and potentially harmful microbes, with different consequences for the host (inflammation versus immune homeostasis) [56]. Moreover, recent data suggested that the context in which the host receptors (PRRs) receive MAMP stimulation determines the quality of the inflammatory and immune response [57, 58]. DSS notoriously disrupts intestinal barrier integrity, allowing commensal microbiota to intestinal epithelium [59, 60]. In Y-DSS pigs, with the increase of potentially harmful microbes, such as *Spirochaetes* [61], microbial ligand signals were received in the presence of intestinal barrier damage and thus might activate PRRs signalling and downstream inflammation-related pathways. In M-DSS pigs, many potentially beneficial symbiotic microbes increased, such as *Lactobacillus*, suggesting that some MAMPs promoted beneficial outcomes. Under such circumstances, PRRs including TLR2, TLR3, TLR4, TLR8, NOD1 and NOD2 exhibited nonactivation compared with M-CON pigs, eliciting tolerant responses. Of course, some PRRs, such as TLR2, were activated via sensing MAMPs. Generally, TLR2 mediates the immune response to gram-positive cell wall peptidoglycan but does not affect the expression of NF-κB and MyD88 [62], shaping beneficial host immune responses. Therefore, compared to Yorkshire pigs, Min pigs might have evolved a more established strategy to modulate the structure of the colonic microbiota and manipulate PRRs pathways, resulting in less inflammation.

The gut microbiota contributes to the development of normal immunity but, when dysregulated, can promote autoimmunity and affect the immunological barrier through effects on lymphocytes [63]. GO analyses showed that the Th17-type immune response was activated, and that a plethora of proinflammatory factors was secreted in Y-DSS pigs. In addition, Th1 and cytotoxic CD8^+^ T-cell (Tc) cytokines (IFN-γ and TNF-α) and Th17 cytokines (IL-17) were increased, suggesting that cell-mediated immunity was promoted in Y-DSS pigs via Th1, Th17 and Tc immune responses. Regarding a possible correlation with microbiota, *Prevotellaceae* genera were increased in Y-DSS pigs, which could stimulate epithelial cell production of IL-6, IL-8 and chemokine ligand 20 (CCL20) and promote the mucosal Th17 immune response and neutrophil recruitment [64]. Stimulation of most TLRs leads to Th1 rather than Th2 differentiation [65]. The levels of some *NLR* and *TLR* genes increased in Y-DSS pigs, which were consistent with the activation of Th1 and Th17-type immune responses. Furthermore, CD8^+^ T-cell levels were markedly increased in the lamina propria of Yorkshire pigs. Although CD8^+^ T cells build a protective barrier against attack by surrounding host cells, their proliferation indicates that numerous pathogens are attacking. Accumulating evidence suggests that colitis is characterised by an expansion of colonic CD8^+^ T cells [66–68]. Distinct from the increase in CD8^+^ T cells in Y-DSS pigs, CD8^+^ T cells remained at steady-state levels in M-DSS pigs, but an increase in CD4^+^ T cells was observed. CD4^+^ T cells reflect immune status; higher levels indicate better immune performance. CD4^+^ T cells can be categorised into Th1 and Th2 cells, which drive different immune responses [69]. GO enrichment analyses of genes under immunity terms revealed CD4 T-cell activation, and acute-phase response was upregulated in Min pigs, indicating acceleration of the end of the inflammatory phase of type-1 immune response and initiation of the Th2-type immune-mediated repair phase. Characterisation and quantification of CD4^+^ T cells and Th2 cytokines may also be robust evidence for supporting our interpretation of the initiation of Th2-type immune-mediated repair phase. To date, investigations found *Clostridia clusters* IV and XVIII [70, 71] promote CD4^+^ regulatory T-cell (Treg) differentiation and *Lactobacillus plantarum* D-alanylated teichoic acid [72] signals through TLR2 to modulate Treg cells, which are negative regulators of inflammation. Moreover, *Lactobacillus reuteri*, together with tryptophan, can reprogram intraepithelial CD4^+^ T cells into tolerant immunoregulatory T cells [73]. The relative abundances of these genera (*Clostridia*, *Lactobacillus*) were increased in M-DSS pigs. Additionally, tryptophan metabolism and TLR2 activation were promoted in M-DSS pigs. These data provided a possible explanation that resident bacteria in Min pigs modulated the immune response to remain tolerant and control excessive inflammatory reactions.

IgA is a major class of immunoglobulins that is produced in mucosal tissues, including in the intestine. In the intestinal lumen, IgA is produced as polymeric IgA at high concentrations [7]. Polymeric IgA is transported via pIgR, which is expressed on intestinal epithelial cells and is released into the intestinal lumen as SIgA [74]. Although the levels of pIgR, SIgA and IgA increased in both M-DSS and Y-DSS pigs, the change was more significant in Y-DSS pigs, suggesting a more robust adaptive immune response in Yorkshire pigs compared with Min pigs. Recent studies have shown a substantial correlation between gut mechanical barrier dysfunction and IBD [75]. The intestinal mechanical barrier is also strongly correlated with the microbiota and its metabolites [76]. On the one hand, numerous bacterial products regulate gut barrier function by activating the TLR and NLR pathways [77]. On the other hand, microbial metabolites (such as SCFAs, secondary bile acids and indole derivatives) produced in the gut directly influence gut barrier integrity by interacting with immune cells [78]. In this study, common functional terms related to intestinal structure and trefoil factors in the maintenance of gastrointestinal epithelium were upregulated in M-DSS pigs, indicating repair of intestinal mucosal damage in Min pigs. In addition, the analyses of colonic microbiota and metabolites and TJ proteins in the mechanical barrier provided a further explanation of the intestinal mechanical barrier dysfunction in Yorkshire pigs and intestinal mechanical barrier restoration in Min pigs. In Y-DSS pigs, depletion of members of *Blautia, Eubacterium*, *Dorea*, *Butyricicoccus* and *Lachnospiraceae* could be associated with the reduction in SCFA concentrations, resulting in obstruction of the epithelial barrier. Decreases in secondary bile acids as a factor may further accentuated gut barrier dysfunction. In M-DSS pigs, enrichment of *Lactobacillus* and promotion of tryptophan metabolism might increase indole derivative levels, which could enhance intestinal barrier function. At the same time, the increases of SCFA production might result from the increase in several potentially beneficial microbes, maintaining colonic OCLN and CLDN levels. In addition, TLR2 activation was increased in M-DSS pigs. TLR2 enhances the integrity of the intestinal epithelial barrier through protein kinase C (PKC) [79]. These data suggested that adjustment of microbial community structures and subsequent metabolite recovery in Min pigs may protect the intestinal barrier in this IBD model.

## Conclusions

**Fig. 10:**
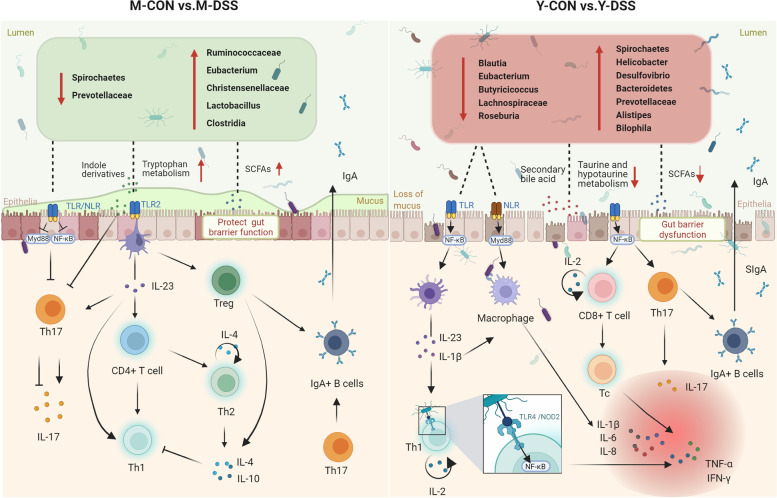
Host-microbiota interaction-mediated resistance to inflammatory bowel disease in Min pigs and Yorkshire pigs. The comparison of the responses of the two breeds to disease and inflammatory immune stimuli showed that colon microbial taxonomies, functions and metabolites and their interactions with host physiological suitability were associated with resistance. Many potentially beneficial microbes, such as *Lactobacillus*, *Clostridia*, *Eubacterium* and several *Ruminococcaceae* and *Christensenellaceae* genera, were increased in diseased Min pigs, which were positively correlated with the improvement of microbial metabolites, including indole derivatives and short-chain fatty acids, and thus may recover the intestinal barrier. In addition, core bacteria altered in abundance, and partial PRR nonactivation was maintained, thereby effectively inhibiting the inflammatory response. The increase in CD4+ T cells in the lamina propria improved the supervision of the host immunity response, contributing to maintenance of Th2-type immune superiority and immune tolerance patterns and controlling excessive inflammatory reactions. In Yorkshire pigs, concentrations of bile acid metabolites and short-chain fatty acids were lower in diseased individuals, associated with the increases in potentially harmful microbes, such as *Bilophila*, *Alistipes* and *Bacteroidetes*, and the depletion of potentially beneficial microbes, such as *Blautia*, *Eubacterium*, *Dorea*, *Butyricicoccus*, *Lachnospiraceae and Roseburia*. The increases in potentially harmful microbes and damaged intestinal barrier in Yorkshire pigs might be a factor in activating PRRs, thereby enriching Toll-like receptor 4 binding, NF-κB signalling and T-helper 1- and 17-type immune responses

Here, an effect of breeds characterised by different disease resistance on host-microbiota interaction was discovered using microbiota sequencing, metabolomics, transcriptomics and other analyses (Fig. 10). Our study suggested that partial PRR nonactivation was maintained in M-DSS pigs with the increases of potentially beneficial microbes and stabilisation of intestinal barrier. Our data also suggested that the increase in CD4^+^ T cells in M-DSS pigs but not in Y-DSS pigs might be relevant to the increases in *Lactobacillus* and tryptophan metabolism, thus retaining Th2-type immune superiority and an immune tolerance pattern. Furthermore, association analyses revealed a positive association between *Lactobacillus*, *Christensenellaceae*, *Eubacterium* and SCFA concentrations, suggesting beneficial action in protecting intestinal barrier in Min pigs. Growing evidence suggests host-microbiota interaction has been linked to IBD disease progression; thus, the results of this study may contribute to screening for core intestinal microbial candidates associated with host health and support a shift in thinking about anti-inflammatory strategies from improving immunity to immunosuppression.

## Supplementary Information

**Additional file 1: Figure S1.** Sankey diagrams of the phylum, family, and genus affiliations of the microbiota in Min pigs and Yorkshire pigs.

**Additional file 2: Figure S2.** Metabolite changes in pig colonic digesta in M-CON vs. M-DSS pigs and Y-CON vs. Y-DSS pigs.

**Additional file 3: Figure S3.** Spearman correlation analysis between gut microbiota and metabolites. The heatmap and correlation network plots show the Spearman correlation coefficients of the comparison groups a,c M-CON vs. M-DSS and b,d Y-CON vs. Y-DSS.

**Additional file 4: Figure S4.** Additional data for RNA-seq analysis. KEGG enrichment analysis of colon genes in M-CON vs. M-DSS and Y-CON vs. Y-DSS.

**Additional file 5: Table S1.** qRT–PCR primer sequences.

## Data Availability

The datasets supporting the conclusions of this article were deposited in the NCBI Sequence Read Archive database under the accession numbers PRJNA797567 (RNA-seq raw data) and PRJNA799232 (microbiota raw sequencing data). All other data are contained within the main manuscript and supplemental files.
