# Supplementary material for: Host-microbiota interaction-mediated resistance to inflammatory bowel disease in pigs
Source: Microbiome. 2022 Jul 30;10:115. doi: 10.1186/s40168-022-01303-1 (PMC9338544; doi:10.1186/s40168-022-01303-1)
Supplement: Supplementary file 2 — Additional file 1: Figure S1. Sankey diagrams of the phylum, family, and genus affiliations of the microbiota in Min pigs and Yorkshire pigs. [file 40168_2022_1303_MOESM2_ESM.docx]

**
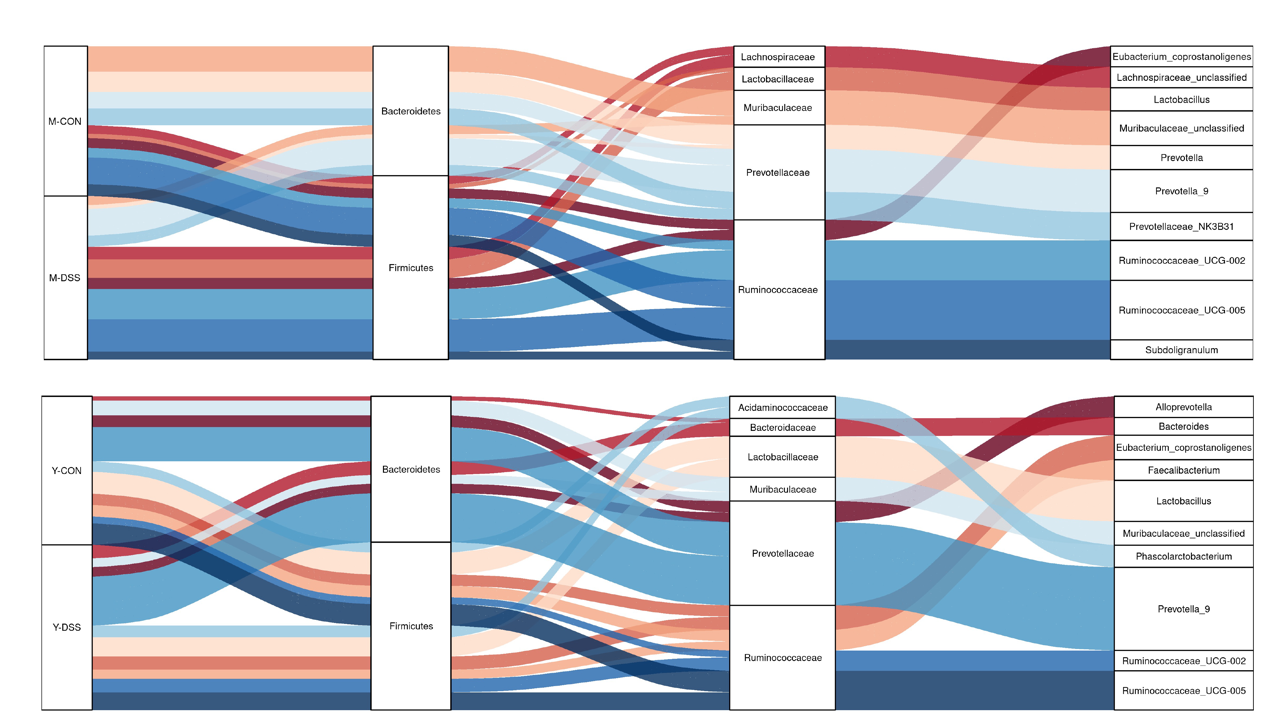
Supplementary Figure 1.** Sankey diagrams of the phylum, family, and genus affiliations of the microbiota in Min pigs and Yorkshire pigs.
