# Supplementary material for: Host-microbiota interaction-mediated resistance to inflammatory bowel disease in pigs
Source: Microbiome. 2022 Jul 30;10:115. doi: 10.1186/s40168-022-01303-1 (PMC9338544; doi:10.1186/s40168-022-01303-1)
Supplement: Supplementary file 3 — Additional file 2: Figure S2. Metabolite changes in pig colonic digesta in M-CON vs. M-DSS pigs and Y-CON vs. Y-DSS pigs. [file 40168_2022_1303_MOESM3_ESM.docx]

**Supplementary Figure 2.** Metabolite changes in pig colonic digesta in M-CON vs. M-DSS pigs and Y-CON vs. Y-DSS pigs.
