# Supplementary material for: Host-microbiota interaction-mediated resistance to inflammatory bowel disease in pigs
Source: Microbiome. 2022 Jul 30;10:115. doi: 10.1186/s40168-022-01303-1 (PMC9338544; doi:10.1186/s40168-022-01303-1)
Supplement: Supplementary file 4 — Additional file 3: Figure S3. Spearman correlation analysis between gut microbiota and metabolites. The heatmap and correlation network plots show the Spearman correlation coefficients of the comparison groups a,c M-CON vs. M-DSS and b,d Y-CON vs. Y-DSS. [file 40168_2022_1303_MOESM4_ESM.docx]

**
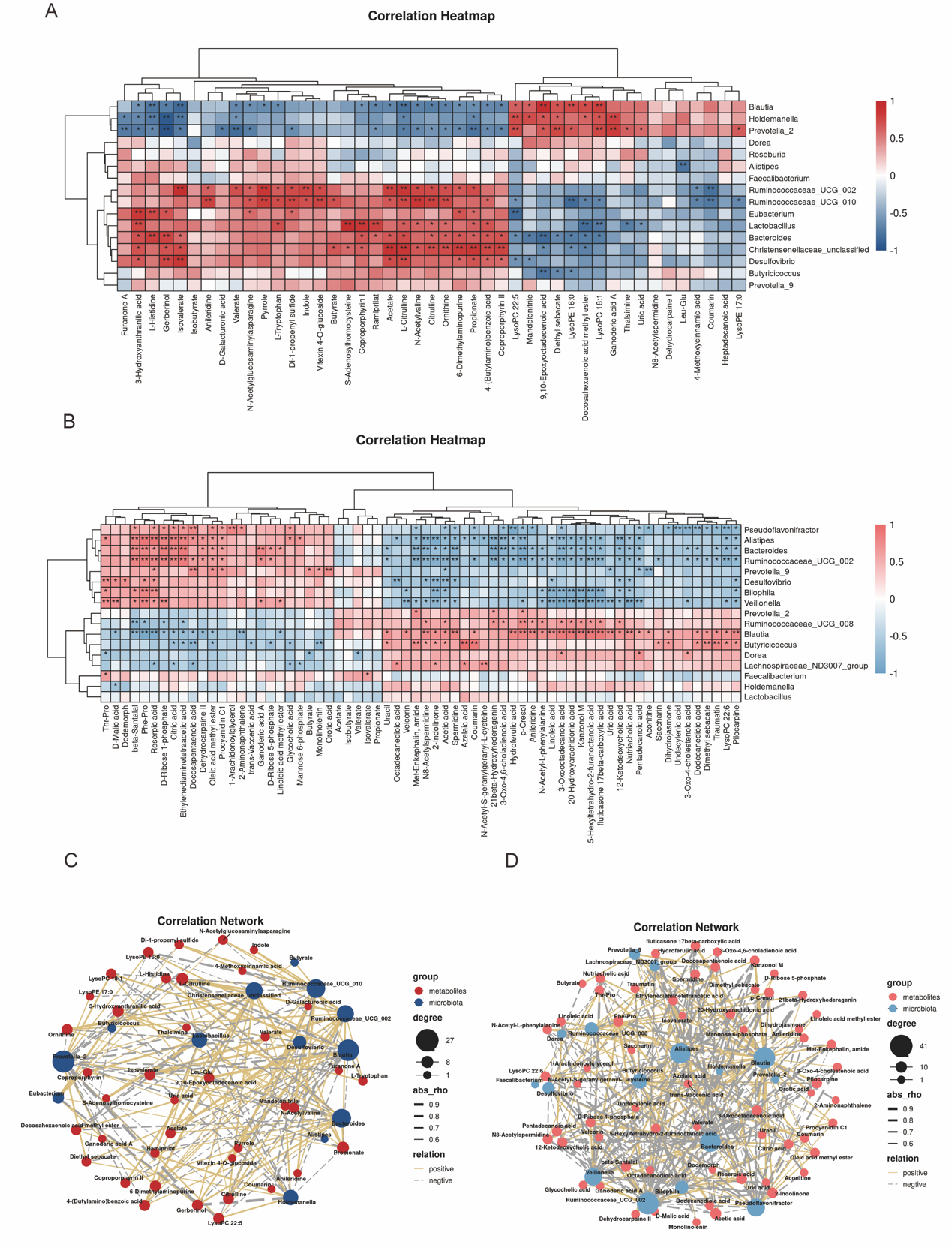
** **Supplementary Figure3.** Spearman correlation analysis between gut microbiota and metabolites. The heatmap and correlation network plots show the Spearman correlation coefficients of the comparison groups **a,c** M-CON vs. M-DSS and **b,d** Y-CON vs. Y-DSS.
