# Supplementary material for: Host-microbiota interaction-mediated resistance to inflammatory bowel disease in pigs
Source: Microbiome. 2022 Jul 30;10:115. doi: 10.1186/s40168-022-01303-1 (PMC9338544; doi:10.1186/s40168-022-01303-1)
Supplement: Supplementary file 5 — Additional file 4: Figure S4. Additional data for RNA-seq analysis. KEGG enrichment analysis of colon genes in M-CON vs. M-DSS and Y-CON vs. Y-DSS. [file 40168_2022_1303_MOESM5_ESM.docx]

**Supplementary Figure4.** Additional data for RNA-seq analysis. KEGG enrichment analysis of colon genes in M-CON vs. M-DSS and Y-CON vs. Y-DSS.
