# Supplementary material for: Host-microbiota interaction-mediated resistance to inflammatory bowel disease in pigs
Source: Microbiome. 2022 Jul 30;10:115. doi: 10.1186/s40168-022-01303-1 (PMC9338544; doi:10.1186/s40168-022-01303-1)
Supplement: Supplementary file 6 — Additional file 5: Table S1. qRT–PCR primer sequences. [file 40168_2022_1303_MOESM6_ESM.docx]

**Supplementary Table1.** qRT–PCR primer sequences

| Genes | Sequences (5' to 3') | Fragments sizes | Gen Bank No. |
| --- | --- | --- | --- |
| *β-actin* | F: GGCACCACACCTTCTACAACGAG | 102 | AY550069.1 |
|  | R: TCATCTTCTCACGGTTGGCTTTGG |  |  |
| *TLR2* | F: ACGTATCCATCAATGAACACTGC | 153 | NM_213761.1 |
|  | R: GTCCGTTAAGGGTGCAGTCA |  |  |
| *TLR3* | F: CAACTGATGCTCCGAAGGGT | 204 | NM_001097444.1 |
|  | R: AGGGTTTGCGTGTTTCCAGA |  |  |
| *TLR4* | F: GACAGCAATAGCTTCTCCAGC | 205 | NM_001113039.2 |
|  | R: GGTTTGTCTCAACGGCAACC |  |  |
| *TLR7* | F:AGACAAGCACTTGACAGCGA | 102 | NM_001097434.1 |
|  | R:TGGAAGGAGGCTGGAGTGAT |  |  |
| *TLR8* | F: TGTCATTGCAGAGTGCACAA | 169 | NM_214187.1 |
|  | R: CAGCTTGGCGTTGTGGTTTAG |  |  |
| *TLR9* | F: CTGGCTCTTCCTGAAGTCCG | 110 | NM_213958.1 |
|  | R: AGTCAGAGTCGTGCAAGTGG |  |  |
| *NOD1* | F: ATGGAGCTGGTGGACTTTGG | 106 | NM_001114277.1 |
|  | R: AAATGGTCTCGCCCTCCTTG |  |  |
| *NOD2* | F: GTGCCTCCCCTCTAGACTCA | 191 | NM_001105295.1 |
|  | R: ACGAACCAGGAAGCCAAGAG |  |  |
| *IL-17* | F: CTCGTGAAGGCGGGAATCAT | 113 | NM_001005729.1 |
|  | R: GGTGTGCTCCGGTTCAAGAT |  |  |
| *pIgR* | F: GAGGGCGAAAAGGTCATCCA | 78 | NM_214159.1 |
|  | R: GCGATAATGCTGTTGGCCTG |  |  |

*TLR2* = Toll like receptor 2; *TLR3* = Toll like receptor 3; *TLR4* = Toll like receptor 4; *TLR7* = Toll like receptor 7; *TLR8* = Toll like receptor 8; *TLR9* = Toll like receptor 9; NOD1 = Nucleotide binding oligomerization domain 1; NOD2 = Nucleotide binding oligomerization domain 2; *IL-17* = Interleukin 17; *pIgR* = Polymeric Immunoglobulin Receptor.
